# The Contribution of Immune Evasive Mechanisms to Parasite Persistence in Visceral Leishmaniasis

**DOI:** 10.3389/fimmu.2016.00153

**Published:** 2016-04-22

**Authors:** Elisangela Oliveira de Freitas, Fabiana Maria de Souza Leoratti, Célio Geraldo Freire-de-Lima, Alexandre Morrot, Daniel Ferreira Feijó

**Affiliations:** ^1^The Jenner Institute, University of Oxford, Oxford, UK; ^2^Instituto de Biofísica Carlos Chagas Filho, Universidade Federal do Rio de Janeiro, Rio de Janeiro, Brazil; ^3^Laboratorio de Biologia do Sistema Imune, Departmento de Imunologia, Instituto de Microbiologia, Universidade Federal do Rio de Janeiro, Rio de Janeiro, Brazil; ^4^Laboratório Integrado de Microbiologia e Imunoregulação, Centro de Pesquisas Gonçalo Moniz, Fundação Oswaldo Cruz (FIOCRUZ), Salvador, Brazil

**Keywords:** leishmaniasis, treatment, *Leishmania donovani*, host protective responses, immune evasive mechanisms

## Abstract

*Leishmania* is a genus of protozoan parasites that give rise to a range of diseases called Leishmaniasis that affects annually an estimated 1.3 million people from 88 countries. *Leishmania donovani* and *Leishmania (L.) infantum chagasi* are responsible to cause the visceral leishmaniasis. The parasite can use assorted strategies to interfere with the host homeostasis to establish persistent infections that without treatment can be lethal. In this review, we highlight the mechanisms involved in the parasite subversion of the host protective immune response and how alterations of host tissue physiology and vascular remodeling during VL could affect the organ-specific immunity against *Leishmania* parasites.

## Introduction

Leishmaniasis is a complex of mammalian neglected tropical diseases, caused by over 20 different parasitic protozoans of genera *Leishmania*. Transmission can occur as zoonotically or anthroponotically, usually by the bite of female by ~30 different species phlebotomine sandflies (1). Three main manifestations can occur that include the cutaneous (CL), mucocutaneous affecting the skin and mucous membranes, and visceral leishmaniasis (VL) (1).

These diseases are endemics in 98 countries, and around 350 million people are at risk. The estimate of annual new cases is around two million (2). VL is a disease that is fatal if untreated; around 500,000 new cases are estimated and 50,000 deaths reported annually (3). The disease is caused by *Leishmania donovani* complex in East Africa and the Indian subcontinent and *Leishmania infantum* in Europe, North Africa, and Latin America (4).

Two different types of VL can occur, which differ in the way of transmission: the zoonotic VL that is transmitted from animal to vector to human and the anthtoponotic VL where transmission from human to vector to human. So, humans are an occasional host and animals, especially dogs, play the role of reservoir of the parasite. In areas of *L. infantum*, the zoonotic VL is found, while in areas of *L. donovani* transmission, anthroponotic VL is found (5).

Visceral leishmaniasis is also known as kala-azar and is characterized by irregular fever, anemia, hepatosplenomegaly, pancytopenia, weight loss, and hypergammaglobulinemia. It is widely confined to East Africa, Indian subcontinent, Brazil, and regions bordering the Mediterranean. Dermal leishmanoid (PKDL) is a macular, maculo-papular, or nodular rash representing a complication of VL that is usually noted after treatment in Sudan and less often in other East African countries and in the Indian Subcontinent. This often affects immunosuppressed individuals in *L. infantum* endemic areas (6). These lesions can appear anywhere on the body, but most commonly occur on the face (7). The interval between treated VL and PKDL is 0–6 months in Sudan and between 6 months to 3 years in India. As the nodular lesions contain many parasites (8), and such cases are the putative reservoir for anthroponotic VL between epidemic cycles, this form of disease is more infectious (6).

For treatment of leishmaniasis few drugs are available at moment. They include: pentavalent antimonium [Sodium atibogluconate (Pentostam^®^)] and meglumine antimoniate (Glucantime^®^), pentamidine, amphotericin B, liposomal amphotericin B, miltefosine, and paramomycin (9, 10). These face limitations for actual treatment, in that most of them require hospitalization that increases the cost, and they are highly toxic (9).

The mechanism of resistance to pentavalent antimonials is the focus of much research; they have been the standard drugs despite their high toxicity (7). Those drugs are not in use now in Bihar State, India, because of the high rate of drug resistance, where more than 65% of previously untreated patients fail to respond or readily relapse. Sodium stibogluconate (Pentostam^®^) and meglumine antimoniate (Glucantime^®^) are still in use elsewhere. Administration is intravenous or intramuscular, and they show the some efficacy when used in equipollent doses (7).

Fatigue, body aches, electrocardiographic abnormalities, raised aminotransferase levels, and chemical pancreatitis are frequently reported secondary effects. Fatal pancreatitis has been reported in patients with VL and HIV infection (11). AmBisome, a liposome formulation of amphotericin B, is the current standard treatment for VL, particularly against *L. donovani* in Bihar, and just one dose treatment was efficient in the treatment in rural public hospitals in Bangladesh (2). The effectiveness of treatment was less against *L. donovani* in East Africa and *L. infantum* in Latin America. The situation was not different when the treatment was with paromomycin that was efficient in the Indian subcontinent but did not work in East Africa (7).

The mechanisms of parasite evasion in VL are not only caused by down modulation of host protective immune response directly. Several reports showed that tissue physiological and vascular remodeling alterations caused by the disease also contribute to parasite replication and persistence. In this review, we discuss: (1) how the parasite subvert the host immune system by infecting specific keys cells and (2) how changes in the tissue structure and physiology could affect organ-specific immunity during VL.

Following the deposition of infective metacyclic promastigotes into the dermis, the skin innate immune system detects invading promastigotes, recruits inflammatory cells to sites of invasion within minutes, and promotes the induction of adaptive immunity (12). Initial sensing of the parasite involves pattern recognition receptors. The host skin immune system initially senses the parasite through pattern recognition receptors and complement receptors present on different cell types including neutrophils, macrophages, dendritic cells (DCs), and natural killer (NK) cells. Several Toll-like receptors (TLRs) such as TLR2, TLR3 (13), TLR4 (14), TLR7 (15), and TLR9 (14) have been shown to contribute to innate sensing and recognition of *Leishmania* by various innate immune cells. This recognition leads to activation of intracellular signaling pathways that are necessary for the initiation of inflammatory responses and control of parasite proliferation by the innate immune response (16).

Neutrophils are essential cells involved in inflammatory response and contribute to phagocytosis and killing of microbial pathogens. However, the precise role of these cells in VL remains to be addressed. McFarlane et al. (17) demonstrated that neutrophil depletion at the beginning of *L. donovani* infection leads to increase in parasite burden in the spleen and bone marrow but not in the liver, enhanced splenomegaly, a delay in the maturation of hepatic granulomas, a decrease in inducible nitric oxide synthase (iNOS) expression within granulomas, and increased levels of IL-4 and IL-10 with significant increase in the ratio of *L. donovani*-specific serum IgG1/IgG2a levels (17).

Although promastigotes are capable of directly invading DCs and macrophages following their deposition by infected sandflies, several TLRs have been shown to contribute to this process and play a vital role in the production of proinflammatory cytokines that are critical for immunity (18). Also activation of inflammasome and production of IL-1β are important for restriction *in vivo* infection with *L. infantum* in murine model (19). Polymorphisms within the human *IL1B* gene are associated with clinical severity of the disease (20).

Experimental studies in mice suggest that the control of VL may be associated with the development of parasite-specific, cell-mediated immune responses involving both CD4^+^ and CD8^+^ T cells (21). These cells produce IFN-γ, which activates infected macrophages, leading to the production of NO and other free radicals that kill the parasites. DCs activate CD8^+^ T cells through mechanisms that involve antigen cross presentation (22). Also, IL-17 producing γδ T cells suppress early control of parasite growth in the liver, and inflammatory monocytes were an important target for the suppressive effects of IL-17 (23).

In VL, both CD4^+^ and CD8^+^ T cells have been implicated in the resistance and healing capacity against *L. donovani*. The production of IFN-γ by helper CD4^+^ T cells and/or CD8^+^ lymphocytes is associated with protection (24). The Th1 and Th17 profile are correlated with infection resolution (25–28) and Th2 response contribute to susceptibility and disease progression (29). High levels of IL-10 are another regulatory cytokine involved immune suppression inducing parasite persistence and chronicity of disease (29). In humans, IL-27 promoted the production of IL-10 and inhibited secretion of IL-17 by CD4^+^ T cells (30). Recently, Ansari et al. (31) showed elevated circulating levels of IL-27 and elevated expression of IL-27p28 and EBI-3 transcripts in VL patients. Owens et al. (32) demonstrated that CD11c^hi^ DCs promote expansion and maintenance of T cells inducing the production of IL-10 and IL-27 *in vivo*.

In infected individuals with active symptoms of VL was observed high levels of IFN-γ and IL-10, the main source of IFN-γ production found in both innate and cellular responses. On the other hand, IL-10 was restricted to CD8^+^ T and B cells (33). In splenic aspirate cells from VL patients, anti-IL-10 antibodies promoted killing of parasite and increased the secretion of IFN-γ and TNF-α in splenic cells *ex vivo* (34). Recently demonstrated in healed ­visceral human ­leishmaniasis patients, CD8^+^ T cells were activated and the granzyme B levels were found increased when compared to naive group and active VL (35).

Suppression of T cell response is thought to be involved in the pathogenesis of VL. Regulatory T cell (Treg)-mediated immune suppression is reported in animal models of *Leishmania* infection. IL-10 receptor blockade mice were resistant to *L. donovani* infection (36). Also, low levels expression of CD40 in DC induced severity to infection by activation of Treg and the production of IL-10 (37). In immunocompromised *aly/aly* mice infected with *L. donovani* CD4^+^ Foxp3^+^ Treg cells were increased in the liver inducing progression of granuloma formation (38). Majumder et al. (14) showed that mice vaccinated with soluble leishmanial antigen (SLA)-pulsed CpG-ODN-stimulated dendritic cells (SLA-CpG-DCs) decreased the number of Treg cells; and consequently, there was low production of TGF-β. Interestingly, IL-17^−/−^ mice infected with *L. infantum* failed to control parasitemia, increasing the proliferation of Treg cells and production of IL-10 (38). In humans, Treg cells produced high levels of IL-10 indicating immune suppression among VL patients (39). This mechanism will be useful to determine drug treatment and disease prognostic.

Studies investigating the immunoregulatory function, CTLA-4 (CD152 – cytotoxic T lymphocyte antigen-4) has a role regulatory in activation of T cells, including Treg cells (40, 41), and PD-1 (programmed cell death-1) is broadly expressed on activated T cells, regulatory T cells, and other hematopoietic cells (42). Administration of monoclonal antibodies against CTLA-4 reduced the burden of parasite in the liver in VL and increased the frequency of IFN-γ and IL-4 producing T cells in the liver (42). Blockade of the PD-1 during *L. infantum* in dogs, CD8^+^ and CD4^+^ T cells recovered functionality and increased reactive oxygen species production of phagocytes (43). Identification of the mechanism of blocking CTLA-4 or PD-1 reverts the downregulation of T cell response to infection. Ligand for the inhibitory receptor PD-1 (B7-H1) constitutively expressed in T cells showed interaction between B7-1: CTLA-4 and the PD-L1 (B7-H1): PD-1 pathways (44). The blockade of B7-H1, the ligand for the inhibitory receptor PD-1, was found to increase survival of CD8^+^ T cells and induce protective immunity (45). Recently, ­HIV-1-coinfected patients with VL Treg cells expressed high levels of CTLA-4, showing impaired immunologic profile explaining persistence and/or relapse of the disease (46).

## Liver, Bone Marrow, and Spleen: Three Organs, Different Immune Responses

One of the hallmarks of VL is hepatosplenomegaly (1, 21, 22, 47). There is a fine line between immune responses that effectively control parasite growth and induce long-term immunity and those that allow parasite persistence and associated disease (29). Thus, differences in splenic and hepatic tissue microenvironments dictate differences in the ability to generate effective immune responses and parasite control in these organs.

The liver is one of the primary target organs in VL. In experimental models of VL, infection in the liver is self-resolving within 2–3 months (22). This resolution of disease is associated with the development of granuloma formation mediated by a Th1 immune response both in humans and dogs as well (48–50). The development of inflammatory granulomas around infected liver macrophages leading to immunity is a T-cell-driven event. This Th1-dominated response is mediated by TLR7, TLR8, TLR9, IL-1, and IL-18 *via* the MyD88 signaling pathway (15). An efficient granuloma formation involves the expression of inducible iNOS by macrophages (22, 51), which is regulated by several pro-inflammatory (Th1) cytokines, such as IL-12, IFN-γ, TNF-α, lymphotoxin, granulocyte/macrophage colony-stimulating factor, IL-2 (52, 53) as well as intact and functional NK and NKT cells (54–56).

*Leishmania* parasites have developed strategies to evade the host immune defenses: invasion of cell types to modulate cell host function to replicate and to downregulate the host immunity for its persistence (24). In a murine model of *L. donovani* infection, liver-resident macrophages (Kupffer cells) infected have a different trancriptomic network profile compared to uninfected Kupffer cells isolated from the same mouse (57). Retinoid X receptor alpha (RXRα) was identified as a key hub within this network, and its pharmacological pertubation with agonists of RXRα enhanced the innate resistance of Kupffer cells to *Leishmania* infection *in vivo* (57). Also Hepatic stellate cells infected *in vitro* and *in vivo* with *L. donovani* produces immunoregulatory cytokines that induces CD4^+^ T cells to become Treg that leads to parasite persitence (58).

Although initially unaffected (due to efficient local immune response), the liver is slowly damaged as the disease progresses (59). Consequently, VL leads to hepatic dysfunction, such as coagulation defects, increased serum concentrations of several liver-specific enzymes, and changes in the cholesterol biosynthesis (60, 61). The liver is the main source of cholesterol biosynthesis in mammals (62) and the decreased serum cholesterol was associated with VL severity and parasite persitence (63, 64). Ghosh et al. (65) identify that the *L. donovani* infection downregulates miR-122 in hepatic tissue, lowering serum cholesterol and increasing parasite burden. The pathology is reversed when hepatic levels of miR-122 are restored with increased serum cholesterol and reduction of liver parasite burden.

In VL, the spleen also becomes chronically infected by mechanisms that are less well understood. In EVL, the spleen becomes enlarged and splenomegaly can account for up to 15% of the body weight of infected mice in as little as 6–8 weeks postinfection (22). The persistence of parasites in the spleen is associated with changes in the splenic lymphoid microenvironment, and concomitant increases in the rate of T-cell apoptosis, decreased responsiveness to leishmanial antigens, and drug resistance (21, 22, 66–68).

The spleen is composed by red (RP) and white pulp (WP), separated by an interface called the marginal zone (MZ). The splenic RP contains macrophages that recycle iron blood from aging red blood cells. The WP is organized similarly to a lymph node, containing T-cell and B-cell follicles. It is in the WP where antigen-specific immune responses are generated (69).

During VL, there is an intense vascular remodeling in the RP and WP (68, 70–72). This vascular change causes disruption to both the gp38^+^ fibroblastic reticular cell network, which guides T cell and DC migration to the T cell zone, and the follicular DC network in the B cell follicles (73, 74). As a consequence, DCs fails to migrate to T cell zone, resulting in an diminishes priming of T cells (73). Dalton et al. (75) showed that by using a receptor tyrosine kinase inhibitor, sunitinib maleate (Sm), vascular remodeling and splenomegaly associated with VL can be blocked, and the pathology can be reversed. The use of Sm alone did not cause a reduction in parasite burden in the spleen; but when combined with conventional antimonial drugs, enhanced leishmanicidal activity with enhanced immune response mediated by CD4^+^ T cells producing IFN-γ and TNF (75).

Bone marrow is also affected during the chronic phase of VL in both patients and experimental models (76, 77). In patients, bone marrow shows moderate to severe megaloblastosis, megakaryocitic hyperplasia, and increased number of plasma cells. All parameters were correlated to parasite load (78). Calvo et al. (79) identified that splenic sequestration and ineffective hematopoiesis appear to be the main etiopathogenetic factors in the bone marrow changes and peripheral cytopenias. This is also observed in experimental models. Lafuse et al. (80) identified increased BFU-E and CFU-E progenitor populations in the spleen and bone marrow and differentially altered erythroid gene expression in these organs. In murine model, there is a correlation in the hematopoietic activity with parasite load in the bone marrow (81). Stromal macrophages are the main target for *L. donovani* infection *in vivo* and *in vitro*; and as a consequence of the selective induction of GM-CSF and TNF-α production, infected stromal macrophages preferentially support increased levels of myelopoiesis (82). Also, Singal and Singh (83) demonstrated that *L. donovani* amastigotes antigen could also induce both *in vitro* and *in vivo* myelopoiesis. If this preferential increase of myelopoeisis may merely serve to increase the number of phagocytes, which are the host cells targets for parasite replication, as well as for increasing the phagocytic uptake of the parasite, further studies are needed to elucidate this question.

## Conclusion and Perspectives

Despite the global public health importance of leishmaniasis, progress in developing vaccines against the disease has lagged because of some key technical hurdles, including the fact that the disease occurs mostly in the world’s poorest countries, and the absence of financial incentives to pharmaceutical companies. Chemotherapy for VL has changed little in 50 years; in areas where drug resistance has yet occurred. The conventional drug treatment still involves parenteral administration of antimonial compounds (Pentostam and Glucantime). Amphotericin B, particularly in liposomal formulation (84), has become the drug of choice in developed countries and where antimony resistance is problematic; but issues of cost and toxicity remain. Also, there are already clinical cases of treatment failure related to liposomal amphotericin B (67, 85). The onset of immunosuppression is a critical event during the progression of VL in a susceptible population. A more comprehensive study would be very helpful for a better understanding about how morpho-physiological tissue alterations and pathogen factors would affect organ-specific immunity during VL. Recently, the use of Systems Biology has been increased (86). Different *in silico* approaches are available for identification of interactions between pathogens and hosts and factors for parasite dissemination and disease progression, as well as to the selection of promising antigens as vaccine candidates, since experimental methods are difficult and time consuming (87, 88). A new approach to develop treatment strategy against VL in resistance cases has to take into account not only by the development of new leishmanicidal drugs but also by the drugs that could reverse the anergic immune response and pathophysiological changes during VL, such as hypocholesterolemia and splenic neovascularization. The use of an anti-vascular therapy (with Sm, for instance) could be an alternative choice to splenectomy in cases of failure treatment for lipossomal Amphotericin B (67, 85).

## Author Contributions

EOF, FML and DFF wrote the paper. DFF and AM made substantial contributions to the conception of the work. CGF-L revised the manuscript.

## Conflict of Interest Statement

The authors declare that the research was conducted in the absence of any commercial or financial relationships that could be construed as a potential conflict of interest.
